# Pea Yogurt Alternatives: Aroma Modification by Starter Selection and Carbohydrate Addition

**DOI:** 10.3390/foods15203723

**Published:** 2026-10-19

**Authors:** Gaiwen Yu, Caimeng Zhang, Xingfei Li, Xiangzhen Kong, Yeming Chen, Yufei Hua

**Affiliations:** 1 College of Agriculture and Biotechnology, Lishui University, Lishui 323000, China; 7200112103@stu.jiangnan.edu.cn; 2 State Key Laboratory of Food Science and Resources, Jiangnan University, 1800 Lihu Avenue, Wuxi 214122, China; cmzhang@jiangnan.edu.cn (C.Z.); lixingfeiangel@163.com (X.L.); xzkong@jiangnan.edu.cn (X.K.); chenyeming19821213@163.com (Y.C.); 3 School of Food Science and Technology, Jiangnan University, 1800 Lihu Avenue, Wuxi 214122, China

**Keywords:** pea yogurt alternatives, starter cultures, carbohydrate supplementation, GC×GC-TOFMS, volatile organic compounds, sensory characteristics

## Abstract

Pea yogurt alternatives integrate the physiological advantages of lactic acid bacteria (LAB) fermentation and the nutritional properties of pea proteins, but their development is limited by the undesirable beany off-flavor of peas. This study aims to improve the aroma profile of LAB-fermented pea milk samples by applying different starter cultures and supplementing carbohydrates. A total of 140 volatile organic compounds (VOCs) were detected using comprehensive two-dimensional gas chromatography coupled with time-of-flight mass spectrometry (GC×GC-TOFMS). Changing starter cultures yielded 26 critical differential VOCs, whereas glucose or lactose supplementation at different levels yielded 27 and 26 critical differential VOCs in fermentations with starters 465 and 061, respectively. The up-regulated critical differential VOCs corresponded to fermentation-generated and biotransformation-generated volatiles, whereas the down-regulated ones were mainly off-odor volatiles in pea milk. Their quantitative patterns were starter culture- and carbohydrate-dependent. Effects of starter selection and carbohydrate addition on sensory characteristics were assessed by summing the relative odor activity values (ROAVs). The creamy, sweet and pungent odors were markedly enhanced by starters used for plant-based yogurt, while glucose or lactose addition at levels of 3% or 5% (*w*/*w*) enhanced creamy odor and inhibited the green odor. These findings provide useful strategies for aroma modification of pea yogurt alternatives.

## 1. Introduction

Peas (*Pisum sativum*) are attracting more attention as ingredients in plant-based foods because of their high nutritional value and suitability as a plant protein source [1,2,3]. Pea yogurt alternatives integrate the physiological advantages of lactic acid bacteria (LAB) fermentation and the nutritional properties of pea proteins [4,5,6]. Currently, numerous commercial starter cultures are employed in dairy yogurt production, while specialized strains tailored for plant-based yogurt fermentation are also being developed [7,8]. Carbohydrates serve as a basic component of cellular biosynthesis and an essential energy supply for microbial growth and proliferation [9]. Several studies have reported supplementing carbohydrates in yogurt and plant-based milk systems to promote starter metabolic activity and influence fermentation performance [8,10].

Flavor contributes significantly to the sensory quality of plant-based yogurts [11,12,13]. The use of peas in yogurt-like foods is limited due to their undesirable sensory attributes known as beany off-flavor [14,15,16]. In our previous study [17], the effects of different pretreatments on yellow peas, including alkali water soaking, dry dehulling and alkali water soaking, boiling water blanching, wet dehulling and alkali water soaking, as well as boiling water blanching-dehulling and acid water soaking, on the volatile components and sensory properties of pea yogurt alternatives were compared, and it was found that a combination of blanching and acid soaking led to the highest sensory scores. However, the above treatments decreased acid formation yield during lactic acid fermentation of pea milk.

LAB fermentation also improves the flavor profile by modifying off-flavor components [18,19,20]. The beany off-odor volatiles in pea milks and other plant-based milks, typically hexanal, (E)-2-heptenal, (E)-2-hexenal and 1-octen-3-ol, can be biotransformed into 1-hexanol, (E)-2-hepten-1-ol, (E)-2-hexen-1-ol, 1-octen-3-one and other volatiles [21,22]. Generally, the latter group (biotransformed) volatiles showed better sensory properties, such as less green and fatty odors but more sweet and fruity odors, than the former group volatiles [23]. It was found that the conversion rate of beany off-odor volatiles was higher for the plant-based yogurt starters than for dairy-based starters [22]. In addition, LAB fermentation of pea and other plant-based milks generates fermentation-related volatiles [18,24]. Among them, carbonyl compounds, namely 2,3-butanedione, acetoin, acetaldehyde, and 2,3-pentanedione, contribute to the yogurt-like aroma [25,26].

The relative odor activity value (ROAV) is widely applied in aroma analysis, as it effectively quantifies the extent to which each compound contributes to the overall sensory aroma profile [22,27,28,29]. Wu et al. [27] investigated four techniques for estimating odor intensity based on the chemical concentration profiles of odor mixtures, including an approach based on summing odor activity values (OAVs). Similarly, Izquierdo Cañas et al. [28] assigned compounds to odor descriptors, and the OAVs assigned to each descriptor were summed to calculate the total intensity for different sensory categories. In fermented soymilks, Yu et al. [29] characterized deductive sensory properties by summing the ROAVs of critical differential aroma-active compounds sharing the same sensory attributes, thereby constructing descriptor-based sensory profiles from volatile data. In a subsequent study, Yu et al. [22] summed the OAVs of off-odor compounds and their biotransformation products associated with each odor descriptor to quantify fermentation-induced changes in the odor characteristics of fermented soymilks.

Although starter- and supplement-dependent aroma changes have been characterized in plant-based yogurt alternatives [30], systematic information on how starter selection and carbohydrate supplementation regulate pea-specific beany off-odor biotransformation and yogurt-like volatile formation remains limited. The main objective of this study is to investigate the possibility of improving the aroma profile of LAB-fermented pea milk samples by applying different yogurt starter cultures and supplementing carbohydrates, and to evaluate the resulting changes in aroma attributes. This study is expected to provide new ideas and methods for aroma modification to improve the product quality of pea yogurt alternatives.

## 2. Materials and Methods

Pea seeds (*Pisum sativum* L.) were procured from a local market in Wuxi, China, in 2024 and stored at 4 °C prior to subsequent experimentation. The n-alkane standards (C7–C40) and the internal standard, 4-methyl-2-pentanol (purity: 99%), were purchased from Sigma-Aldrich Chemical Co. (St. Louis, MO, USA). Prior to sample analysis, the internal standard was diluted with high-performance liquid chromatography (HPLC)-grade water to a final concentration of 60 mg/L. Five commercial direct-vat-set (DVS) starter cultures were used for pea milk fermentation, including three cultures formulated for dairy yogurt (465, 485, and 900) and two cultures for plant-based fermentation (061 and 022). The declared microbial compositions of the starter cultures are summarized in Table 1. All cultures were obtained from Danisco-DuPont (Dangé-Saint-Romain, France) and were used as supplied. Glucose and lactose (food grade) were purchased from Zhejiang Yinuo Biotechnology Co., Ltd. (Hangzhou, China).

### 2.1. Preparation of Fermented Samples

Pea milk was prepared directly from the starting material without pretreatments to avoid loss of fermentation ability [17]. Peas were thoroughly washed and subsequently immersed in water for 12 h at (25 ± 0.1 °C). A slurry was prepared by mixing the soaked peas with water at a dry pea-to-water mass ratio of 1:4, followed by homogenization using a high-speed blender for 5 min. The resultant slurry was filtered through a 100-mesh sieve to eliminate solid residues, and the filtrate was centrifuged at 4 °C for 15 min at 1500× *g*. The resulting supernatant was then subjected to heat treatment (95 °C, 15 min) to inactivate inherent enzymes. After cooling to room temperature (25 ± 0.1 °C), the supernatant was designated as pea milk.

The protein content and pH of the pea milk were adjusted to 2.5% (*w*/*w*) and 7.3, respectively, by adding deionized water and 0.1 mol/L NaOH solution. For the starter-selection experiment, five starter cultures (465, 485, 900, 061, and 022) were evaluated. For the carbohydrate supplementation experiments, starter cultures 465 and 061 were used, and glucose or lactose was added to the pea milk at 1, 3, or 5% (*w*/*w*), with unsupplemented pea milk serving as the 0% control. The resulting pea milk formulations were then pasteurized at 95 °C for 10 min, followed by cooling to room temperature. The corresponding yogurt starter cultures were inoculated into the pea milk formulations at 0.043 g/kg. Fermentation was carried out at 42 °C until the pH decreased to 4.70. The fermented pea milk samples were stored at −80 °C for further analysis.

### 2.2. VOC Analysis by GC×GC-TOFMS

#### 2.2.1. Extraction of VOCs

Three grams of sample was precisely weighed into a headspace vial (20 mL), to which sodium chloride (0.8 g) and 40 μL of 4-methyl-2-pentanol (60 mg/L), as an internal standard, were added. The sample vial was then equilibrated at 55 °C with magnetic stirring at 500 rpm for 5 min, followed by a solid-phase microextraction process. A divinylbenzene/carboxen/polydimethylsiloxane (DVB/CAR/PDMS) extraction fiber (50/30 μm, 2 cm; Supelco, Bellefonte, PA, USA), previously conditioned at 250 °C for 1 h, was inserted into the headspace of the vial and exposed at 55 °C for 30 min to allow adsorption. Upon completion of adsorption, the fiber was immediately inserted into the gas chromatograph inlet for thermal desorption for 5 min at 230 °C.

#### 2.2.2. GC×GC-TOFMS Analytical Conditions

Comprehensive two-dimensional gas chromatography coupled with time-of-flight mass spectrometry (GC×GC-TOFMS) analysis was conducted as described by Yang et al. [31] with slight modifications. The analytical system comprised a cryogenic jet modulator (LECO Corporation, St. Joseph, MI, USA), a gas chromatograph (Agilent 7890A; Agilent Technologies, Santa Clara, CA, USA), and a LECO Pegasus 4D time-of-flight mass spectrometer. A DB-WAX column (30 m × 0.25 mm × 0.25 μm; Agilent Technologies) was employed for the first dimension, whereas a DB-17 column (2.0 m × 0.1 mm × 0.1 μm; Agilent Technologies) was used for the second dimension. High-purity helium (≥99.999%) was used as the carrier gas at a constant flow rate of 1 mL/min with a split ratio of 5:1. The modulation period was 3 s. The oven temperature was programmed as follows: kept at 40 °C for 1 min, increased to 160 °C at a rate of 3 °C/min, held for 2 min, then raised to 250 °C at 10 °C/min and held for 5 min. A detector voltage of 1780 V and an ionization energy of 70 eV were applied. The transfer line and ion source were held at 270 °C and 230 °C, respectively, with a mass scan range of *m*/*z* 29–400.

#### 2.2.3. Identification and Relative Quantitative Analysis of VOCs

Compounds were identified by matching their mass spectra with spectra in the NIST17.L mass spectral library and comparing their experimental retention indices (RIs) with literature values. The RIs were calculated using a homologous series of C7–C40 n-alkanes as described by Jia et al. [32]. The relative content of each volatile organic compound (VOC) was semi-quantitatively determined using the internal standard method based on the ratio of its characteristic ion peak area to that of the internal standard and the known amount of internal standard added.

### 2.3. Calculation of Relative Odor Activity Values

ROAV was determined as the ratio of the relative concentration of each VOC to its corresponding odor threshold in water, as shown in Equation (1):

(1) $$\mathrm{ROAV}=\frac{C_{i}}{{\mathrm{OT}}_{i}}$$

where C_i_ denotes the relative content of each VOC and OT_i_ represents its corresponding odor threshold. Odor threshold data were obtained from “Odour Thresholds: Compilations of Odour Threshold Values in Air, Water and Other Media” [33].

### 2.4. Sensory Evaluation and Deductive Sensory Characteristics

Twelve panelists (six males and six females, aged 20–55 years) were selected and provided with appropriate training and supervision in accordance with ISO 8586:2012 [34]. The specific experimental procedure was as follows: first, 20 g of fermented pea milk was placed in a 50 mL plastic cup, and sensory analysis was performed in an environment free of odor interference. The panelists thoroughly discussed and analyzed the potential aroma descriptors reported in studies of dairy- and plant-based fermented products [29,35,36,37,38,39,40]. Based on these discussions, six aroma descriptors, namely creamy, fatty, pungent, fruity, sweet, and green, were selected to characterize the overall aroma profile of the fermented pea milk. In general, each VOC is associated with one or more sensory attributes according to the Flavornet and The Good Scents Company databases (www.flavornet.org/flavornet.html (accessed on 20 May 2026) and http://www.thegoodscentscompany.com/ (accessed on 20 May 2026)). To characterize the aroma profile of fermented pea milk, the ROAVs of critical differential aroma-active VOCs (critical differential VOCs with ROAV > 1) sharing the same sensory attribute were summed [22,27,28,29]. If a VOC possessed multiple attributes, the ROAV was equally assigned to each attribute. The sensory evaluation was therefore qualitative, without intensity rating on a scale; the intensity of each aroma attribute was expressed as the sum of ROAVs, which is dimensionless. The resulting aroma attribute profiles reflect the calculated aroma potential rather than a direct human sensory evaluation and are referred to as deductive sensory characteristics, following our previous study [29]. In addition, hierarchical cluster analysis (HCA) was performed on the calculated sensory attribute values to classify the sensory characteristics of the fermented pea milk samples. The resulting clusters were further ranked according to their overall sensory attribute profiles, with relatively higher creamy odor and lower green and fatty odors assigned lower grade numbers, indicating more desirable sensory characteristics.

### 2.5. Statistical Analysis

Subsequent statistical analysis was performed using differences in relative VOC concentrations between fermented pea milk samples and unfermented pea milk as variables. Principal component analysis (PCA), HCA, and partial least squares discriminant analysis (PLS-DA) were carried out using SIMCA 14.1, with data preprocessed using Pareto scaling. HCA was performed using Euclidean distance and complete linkage. Duncan’s multiple range test (significance level *p* = 0.05) was conducted using SPSS 26.0. Critical differential VOCs, representing those VOCs that were markedly changed by starter selection or carbohydrate supplementation, were screened according to the criteria: variable importance in projection (VIP) > 1.0 in the PLS-DA models and one-way analysis of variance (ANOVA, *p* < 0.05). All fermented pea milk samples were prepared in triplicate from independent biological replicates under identical fermentation conditions.

## 3. Results and Discussion

### 3.1. Unsupervised Overall Assessment of the Volatile Components

A total of 140 VOCs were identified in the unfermented and fermented pea milk samples, including 31 aldehydes, 29 ketones, 33 alcohols, 10 acids, 13 esters, and 24 other compounds. The unfermented pea milk was dominated by aldehydes and alcohols, which accounted for 42.9% and 26.6% of the total VOC content, respectively. After fermentation, the proportion of aldehydes decreased to 20.6–25.3%, whereas those of ketones and alcohols increased to 20.8–24.9% and 31.2–34.5%, respectively. To compare the VOC profiles among the fermented samples, multivariate statistical analyses were further performed.

#### 3.1.1. Fermentation by Different Starter Cultures

**Figure 1:**
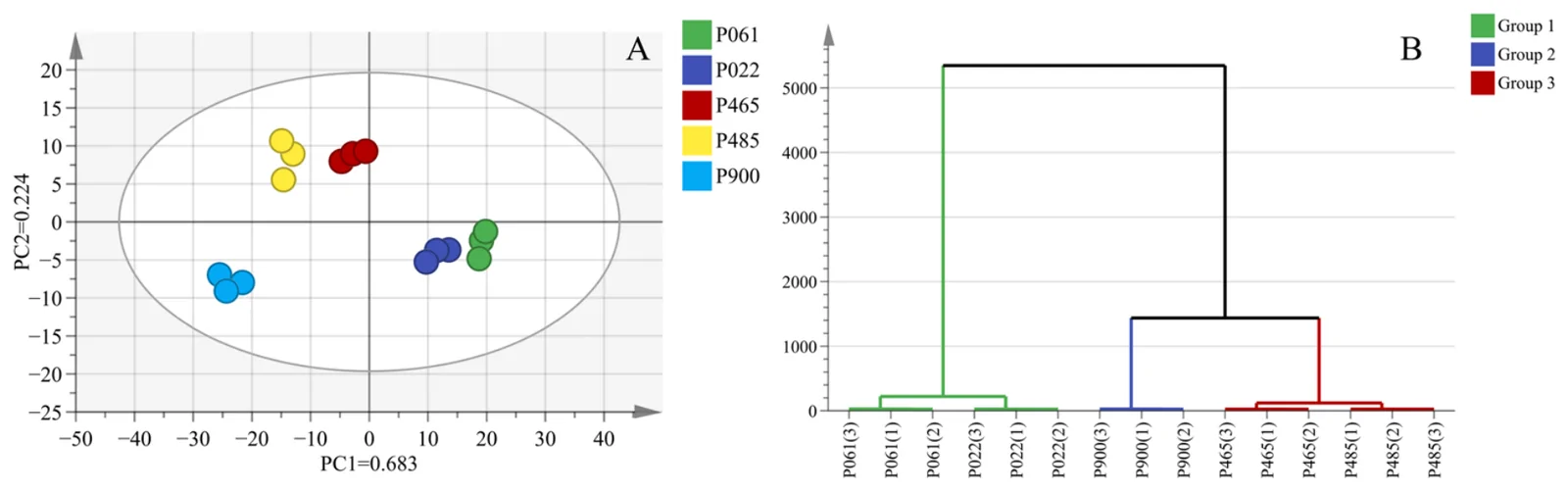
Principal component analysis (PCA) score plot (**A**) and hierarchical cluster analysis (HCA) dendrogram (**B**) of volatile organic compounds (VOCs) in pea milk fermented with different starter cultures. In this and subsequent figures, the multivariate analyses of VOCs were based on the differences in relative VOC concentrations between fermented and unfermented pea milk; in the score plots, each point represents one biological replicate, the values in the axis labels are the explained variances of the first two components, and the ellipses indicate the 95% Hotelling’s T^2^ confidence regions; in the dendrograms, HCA was based on Euclidean distance and complete linkage, the y-axis shows the linkage distance, and the numbers in parentheses are replicate numbers; and P465, P485, P900, P061, and P022 represent pea milk fermented with the corresponding starter cultures.

HCA and PCA showed distinct separation between pea milk samples fermented using different starter cultures, as illustrated in Figure 1A,B. The cumulative explained variance of the first two principal components (PC1 and PC2) in LAB-fermented pea milk samples reached 90.7% (Figure 1A). Figure 1B presents the HCA results of VOCs in pea milk samples fermented with five different starters. The results showed that starter 900 formed a separate cluster, whereas 465 and 485 were grouped, and 061 and 022 constituted another cluster. These results indicated that different starter cultures substantially affected the VOCs in fermented pea milk samples.

#### 3.1.2. Supplementation of Carbohydrates

**Figure 2:**
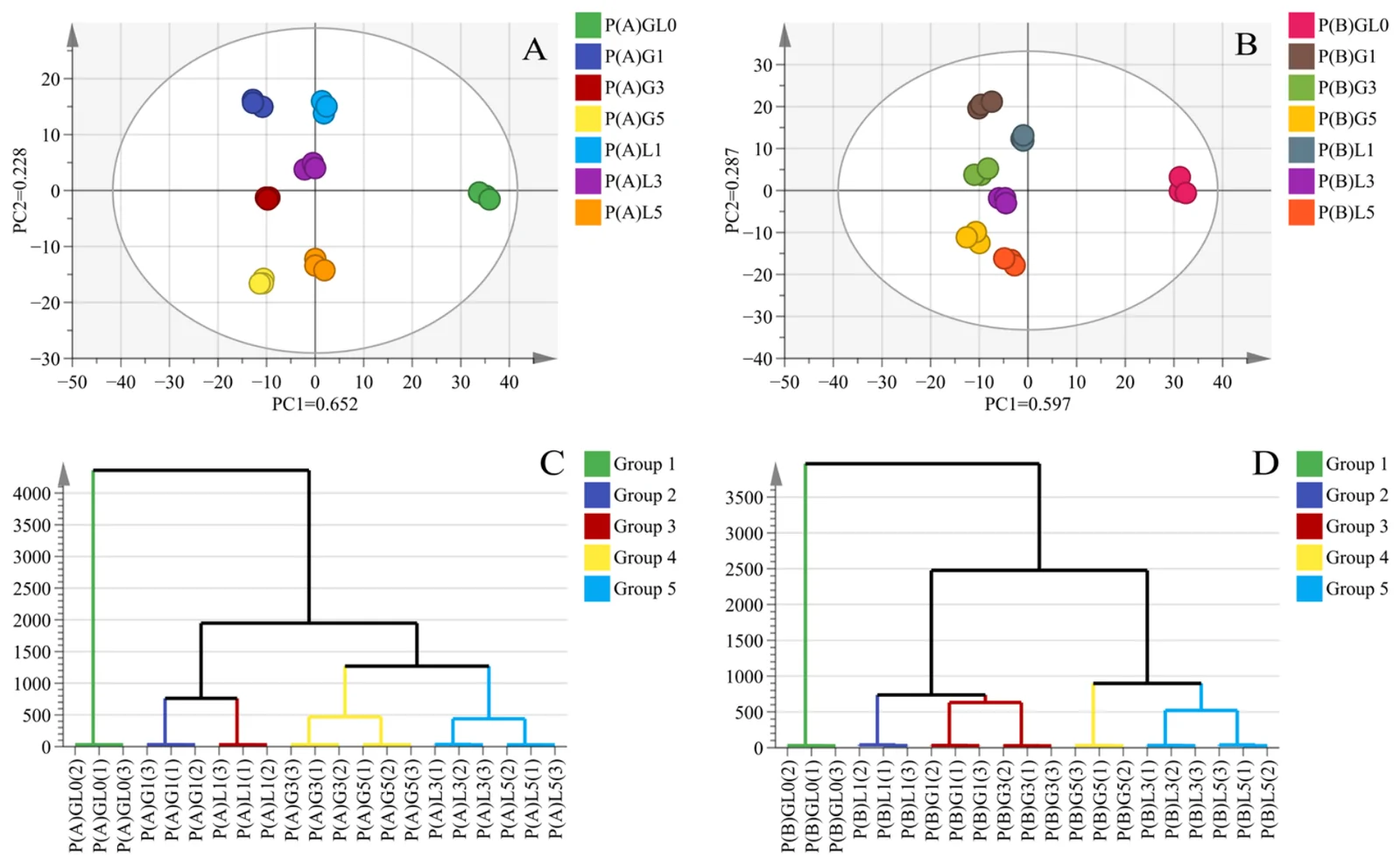
PCA score plots (**A**,**B**) and HCA dendrograms (**C**,**D**) of VOCs in pea milk supplemented with different levels of glucose or lactose and fermented with starters 465 (**A**,**C**) and 061 (**B**,**D**). In this and subsequent figures, P(A) and P(B) represent pea milk fermented with starters 465 and 061, respectively; G and L refer to glucose and lactose; numbers 0/1/3/5 indicate carbohydrate addition levels (%, *w*/*w*); and GL0 denotes no added carbohydrate.

As shown in Figure 2A,B, PCA revealed notable variations in the VOCs of 465- and 061-fermented pea milk samples with varying glucose or lactose supplementation levels. The variance contributions of PC1 and PC2 were 65.2% and 22.8% for culture 465, and 59.7% and 28.7% for culture 061, with cumulative explained variances reaching 88.0% and 88.4%, respectively.

As shown in Figure 2C,D, HCA classified the 465- and 061-fermented samples into five clusters each. For starter 465, the samples with no added carbohydrate, 1% glucose, and 1% lactose each formed a separate cluster, whereas those with 3% and 5% glucose clustered together, as did those with 3% and 5% lactose. For starter 061, the samples with no added carbohydrate and those with 1% lactose each formed a separate cluster, the samples with 1% and 3% glucose clustered together, those with 5% glucose formed a separate cluster, and those with 3% and 5% lactose clustered together. Clearly, the effects of glucose and lactose addition on the volatile components were level-dependent regardless of the starter culture used. A previous study showed that carbohydrate supplementation altered the off-odor volatile profile of fermented soymilk [41].

### 3.2. Screening of Critical Differential VOCs

#### 3.2.1. Fermentation by Different Starter Cultures

**Figure 3:**
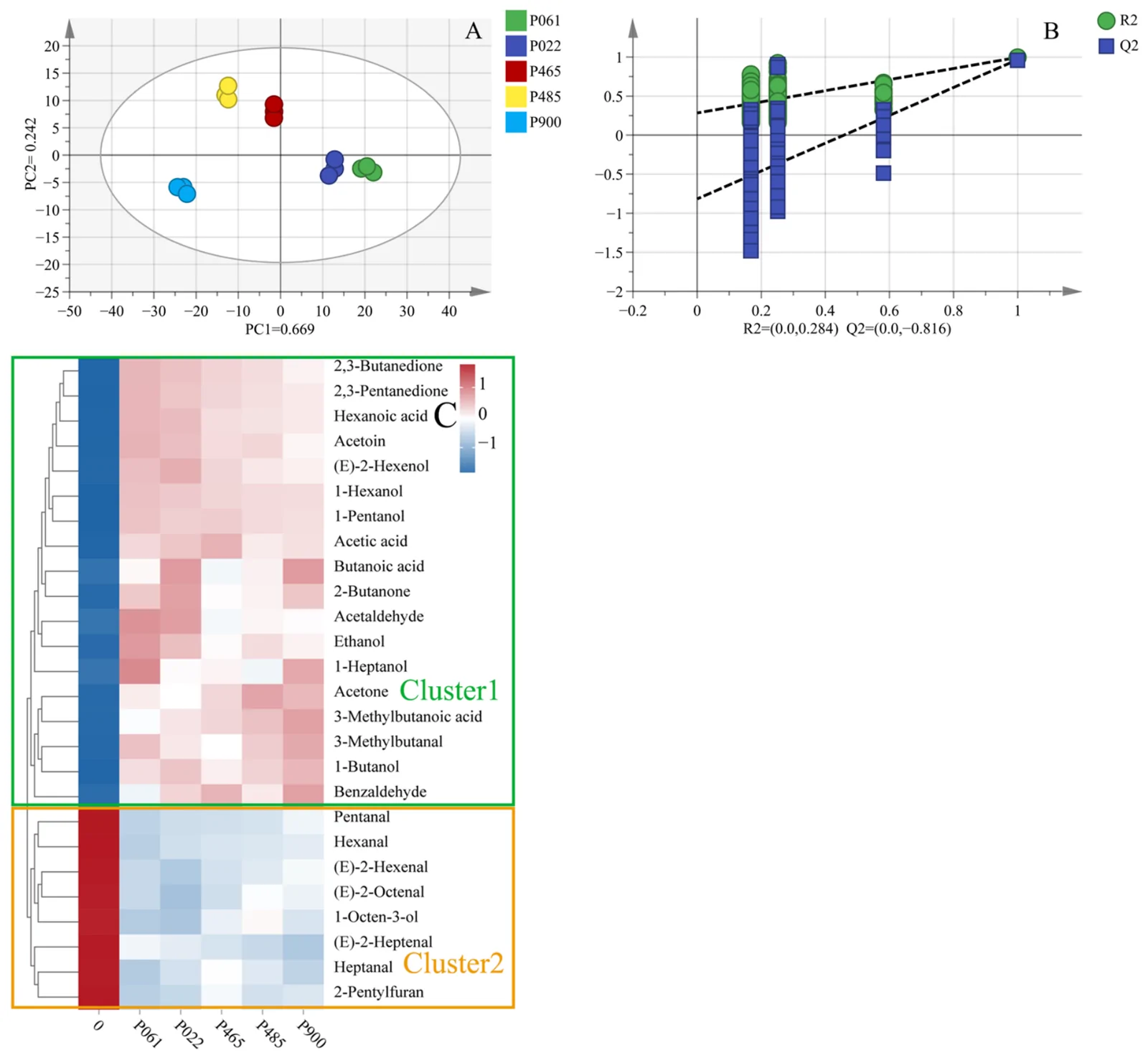
Analysis of the effects of different starter cultures on critical differential VOCs in fermented pea milk samples. (**A**) Partial least squares discriminant analysis (PLS-DA) score plot of the VOC profiles of pea milk fermented with the five starter cultures. (**B**) Permutation test (*n* = 200) of the PLS-DA model in (**A**). (**C**) Heatmap of the 26 critical differential VOCs, with rows clustered by HCA; the VOCs in Cluster 1 and Cluster 2 were up-regulated and down-regulated, respectively, relative to unfermented pea milk (0). In this and subsequent figures, critical differential VOCs are those with variable importance in projection (VIP) > 1.0 and *p* < 0.05 in one-way analysis of variance; in the permutation plots, the R^2^ and Q^2^ values of the permuted models (y-axis) are plotted against the correlation between the permuted and original class labels (x-axis), the points at x = 1.0 represent the original model, and the intercepts of the dashed regression lines are given below the x-axis; and in the heatmaps, colors indicate scaled values (red, high; blue, low).

To investigate the effects of different starters on critical differential VOCs in fermented pea milk samples, a PLS-DA model was used. The PLS-DA model was constructed from the volatile profiles of pea milks fermented by different starters, which revealed clear separation in the score plot, with R^2^ and Q^2^ values both exceeding 0.9, indicating good model fit and predictive ability (Figure 3A). To assess potential model overfitting, permutation tests (*n* = 200) were performed (Figure 3B). All permuted R^2^ and Q^2^ values were lower than the original values, the R^2^ intercept was 0.284, and the Q^2^ intercept was negative (−0.816), indicating that the model was valid and not overfitted. As presented in Figure 3C, 26 critical differential VOCs (VIP > 1.0, *p* < 0.05) were identified in the pea milks fermented by five different starters.

The critical differential VOCs were divided into two clusters using HCA based on Euclidean distance and complete linkage. Cluster 1 represented volatile compounds that were up-regulated relative to unfermented pea milk, including LAB fermentation-generated volatiles (acetaldehyde, 2,3-pentanedione, 2,3-butanedione, acetoin, acetone, and acetic acid) and biotransformation-generated volatiles (1-hexanol, hexanoic acid, (E)-2-hexenol, and 1-pentanol). Cluster 2 represented pea milk off-odor volatiles, including hexanal, pentanal, (E)-2-heptenal, and (E)-2-hexenal, which were down-regulated relative to unfermented pea milk due to biotransformation during fermentation. Among the different samples, critical differential VOCs from starter 061 fermentation showed relatively higher levels of fermentation-generated volatiles such as 2,3-butanedione, acetoin, acetaldehyde, ethanol and 2,3-pentanedione along with relatively lower levels of off-odor compounds (e.g., hexanal, pentanal and heptanal), indicating a VOC profile characterized by higher levels of typical yogurt volatiles and lower residual levels of off-odor compounds. Starter 022 showed relatively high levels of non-typical yogurt volatiles such as acetic acid and 2-butanone, whereas starter 900 showed relatively high levels of acetone and 3-methylbutanal together with comparatively higher residual levels of hexanal and (E)-2-hexenal. These starter-dependent differences are consistent with previous reports that LAB fermentation can decrease aldehyde-type beany volatiles while increasing ketones and other fermentation-related compounds in pea and soy matrices [42,43,44]. Because all five commercial starters contained *Streptococcus thermophilus* and *Lactobacillus delbrueckii* subsp. *bulgaricus* but their relative proportions were unknown, the specific basis for the differences among starters could not be determined. Nevertheless, differences in the undisclosed strain proportions may partly account for the variation in VOC profiles among starters, particularly among 465, 485, and 900, which had identical declared species. This may be because individual strains differ in their contributions to the formation of typical yogurt volatiles and the biotransformation of off-odor aldehydes such as hexanal. The declared species of starters 061 and 022 differed only in *Lacticaseibacillus paracasei* (061) and *Lactiplantibacillus plantarum* (022); in addition to this difference, different strain proportions may have contributed to the higher levels of typical yogurt volatiles and lower residual levels of off-odor compounds in the 061-fermented samples.

#### 3.2.2. Supplementation of Carbohydrates

**Figure 4:**
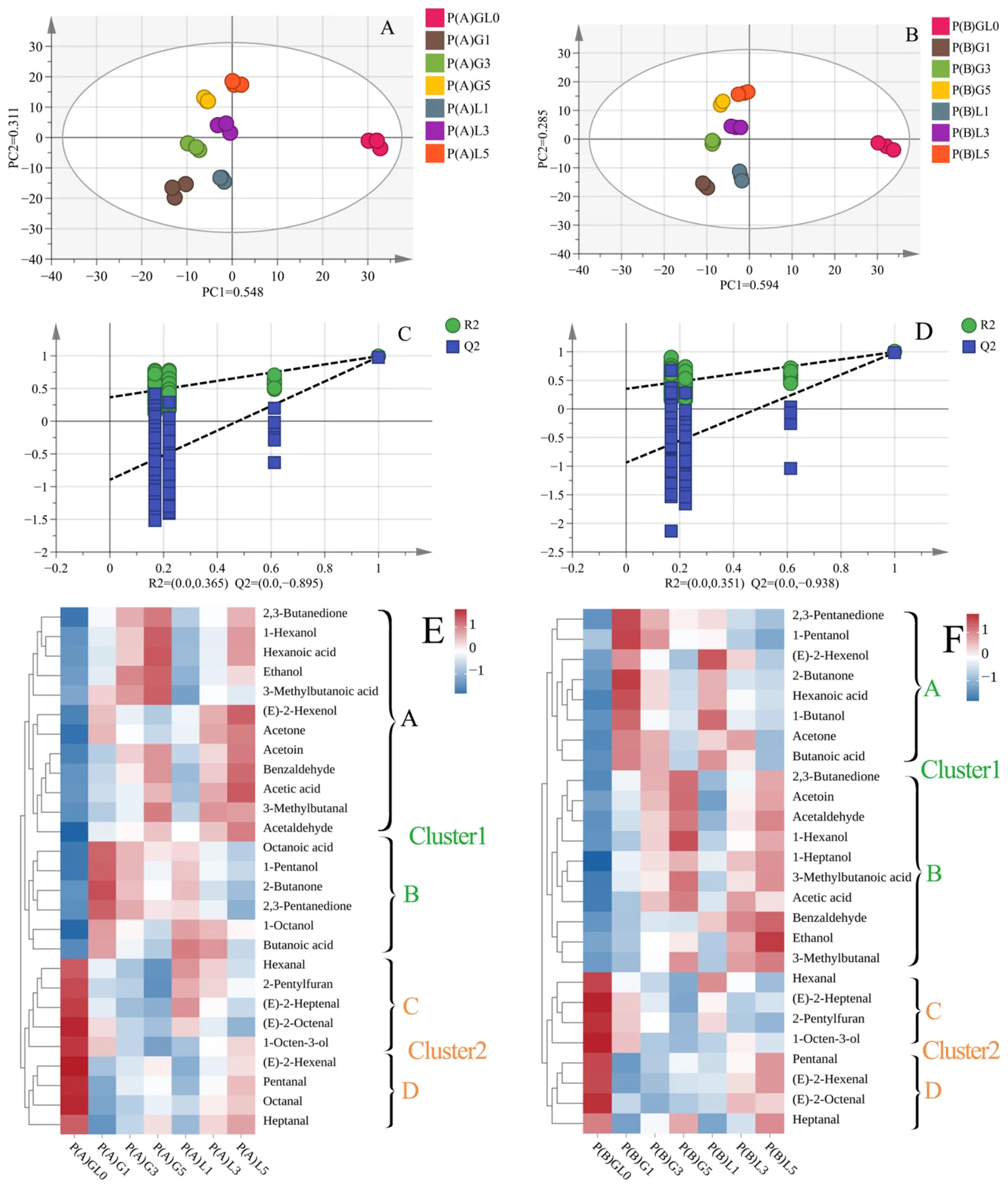
Effects of different types and concentrations of added carbohydrates on the critical differential VOCs in fermented pea milk samples. (**A**,**B**) PLS-DA score plots of the VOC profiles of samples fermented with starters 465 (**A**) and 061 (**B**). (**C**,**D**) Permutation tests (*n* = 200) of the PLS-DA models in (**A**) and (**B**), respectively. (**E**,**F**) Heatmaps of the 27 and 26 critical differential VOCs for starters 465 (**E**) and 061 (**F**), with rows clustered by HCA; the VOCs in Cluster 1 (groups A and B) and Cluster 2 (groups C and D) were up-regulated and down-regulated, respectively, relative to the unsupplemented samples (GL0).

Carbohydrate-supplemented pea milks were fermented with starters 465 and 061, respectively, and the critical differential VOCs in the fermented products were analyzed using PLS-DA models, as shown in Figure 4. The R^2^ and Q^2^ values of both models were greater than 0.8, indicating good model fit and predictive ability. To assess potential model overfitting, permutation tests were carried out under the corresponding conditions (*n* = 200). All permuted R^2^ and Q^2^ values were lower than the original values, the R^2^ intercepts were 0.365 and 0.351, and the Q^2^ intercepts were negative (−0.895 and −0.938 in Figure 4C,D, respectively), indicating that both models were valid and not overfitted. In 465-fermented pea milk samples, differences in carbohydrate addition resulted in 27 critical differential VOCs (VIP > 1.0, *p* < 0.05) while there were 26 critical differential VOCs in 061-fermented pea milk samples. The critical differential VOCs were composed of 5 ketones each, 10 and 9 aldehydes, 6 and 7 alcohols, 5 and 4 acids, and 1 other compound each. Among them, 24 critical differential VOCs, including 2,3-butanedione, (E)-2-hexenal, acetoin, acetaldehyde, and (E)-2-heptenal, were common to both starter 465- and 061-fermented pea milk samples. Octanoic acid, 1-octanol, and octanal were unique to the critical differential VOC set identified for starter 465 fermentation, whereas 1-butanol and 1-heptanol were unique to that identified for starter 061 fermentation.

As shown in Figure 4E,F, the critical differential VOCs were grouped into four distinct groups according to Euclidean distance and HCA, with up-regulated and down-regulated volatiles each forming two separate groups. The up-regulated volatile compounds (groups A and B) mainly consisted of fermentation-generated volatiles such as acetaldehyde, 2,3-pentanedione, 2,3-butanedione, acetoin, and acetone, as well as biotransformation products (e.g., 1-hexanol, hexanoic acid, (E)-2-hexenol, and 1-heptanol). By contrast, the down-regulated volatile compounds (groups C and D) mainly consisted of off-odor components inherent in pea milk, such as hexanal, 1-octen-3-ol, (E)-2-hexenal, and (E)-2-heptenal, which were partially biotransformed during the fermentation process. Therefore, carbohydrate addition modulated both yogurt aroma generation and off-odor biotransformation processes. Similar carbohydrate-dependent changes in critical differential VOCs have been reported in fermented soymilk, where glucose and lactose supplementation altered both fermentation-related and substrate-derived volatiles [29].

### 3.3. Deductive Sensory Characteristics

#### 3.3.1. Fermentation by Different Starter Cultures

**Figure 5:**
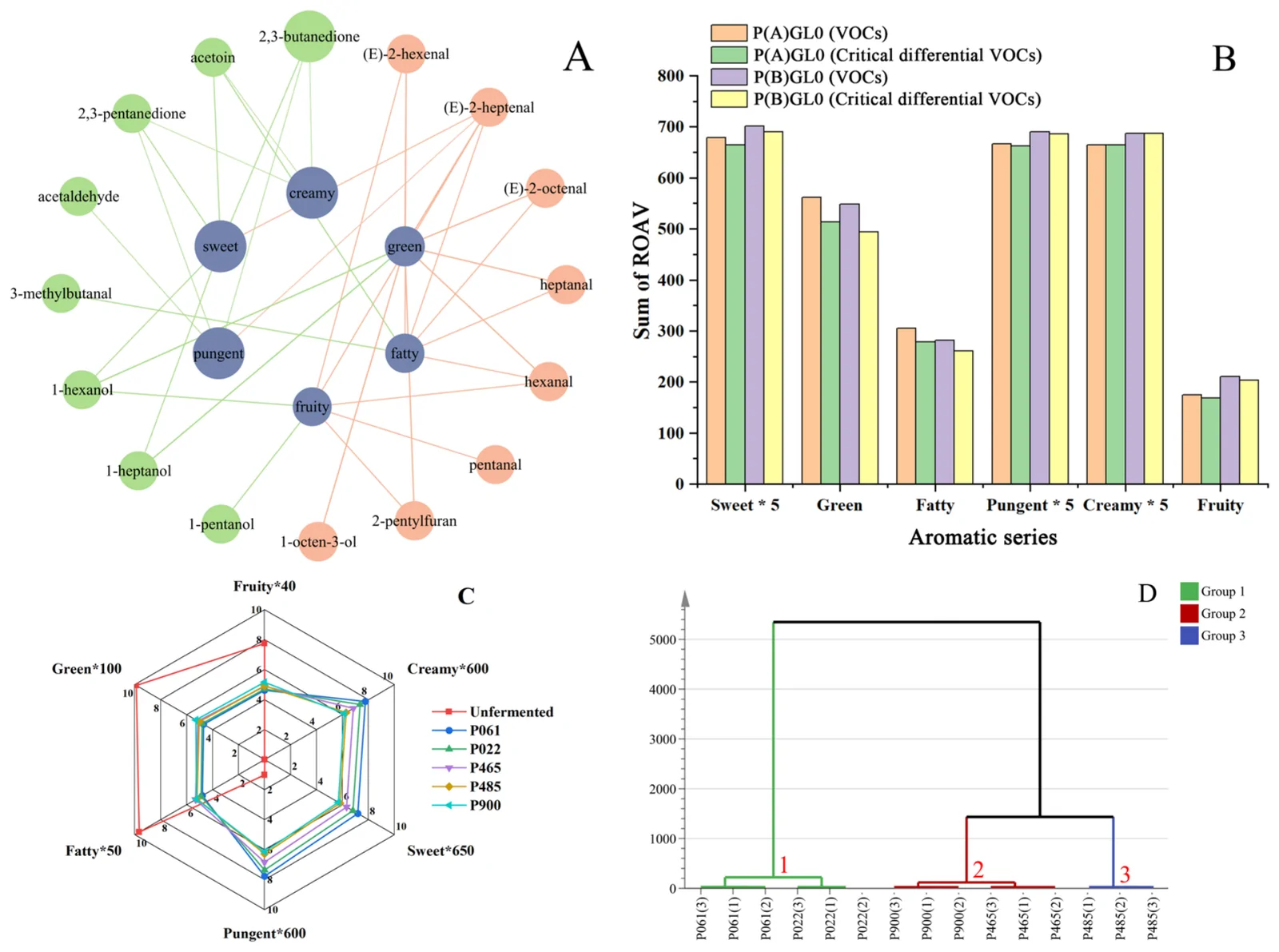
Deductive sensory characteristics of fermented pea milk samples regulated by different starter cultures. (**A**) Association network between sensory attributes and critical differential aroma-active VOCs in pea milk fermented with starter 061. The blue nodes indicate sensory attributes, and the size of the blue nodes corresponds to the sum of the relative odor activity values (ROAVs) of the relevant aroma-active compounds (ROAV > 1). The external nodes represent critical differential aroma-active VOCs, with green and orange denoting fermentation- and biotransformation-generated VOCs and pea milk off-odor VOCs, respectively. The thickness of the lines is proportional to the number of edges of the sensory attributes. (**B**) Bar chart of the summed ROAVs of each sensory attribute in P(A)GL0 and P(B)GL0; the sums were calculated from all VOCs with ROAV > 1 and from the critical differential aroma-active VOCs. (**C**) Radar plot of the summed ROAVs of the sensory attributes in unfermented and fermented pea milk; the sums were calculated from the critical differential aroma-active VOCs. (**D**) HCA dendrogram based on the summed ROAVs of the sensory attributes in pea milk fermented with the five starter cultures. In this and subsequent figures, the number following the asterisk in an attribute label (e.g., Sweet*5) indicates the factor by which the values were divided for plotting, and the red numbers in the dendrograms indicate the grades of the clusters, with lower numbers representing more desirable sensory characteristics (higher creamy odor and lower green and fatty odors).

The effect of starter cultures on sensory properties of fermented pea milk samples was illustrated using starter 061 as an example. Among 26 critical differential VOCs, 16 were aroma-active compounds with ROAV > 1. Among them, 1-octen-3-ol, 2,3-butanedione, hexanal, and 2-pentylfuran had ROAVs exceeding 50, while acetoin, 2,3-pentanedione, (E)-2-octenal, and pentanal had ROAVs ranging from 10 to 50. Figure 5A presents the sensory attributes of these compounds. Notably, a single volatile can exhibit multiple sensory attributes. For example, 2,3-butanedione shows sensory attributes of creamy, sweet and pungent. Conversely, multiple volatiles were associated with a single sensory attribute; for example, green odor was associated with 9 compounds.

For each aroma descriptor, the ROAVs corresponding to the VOCs with ROAV > 1 and to the critical differential aroma-active VOCs were summed up separately and the results are shown in Figure 5B. The two summed ROAVs for aroma descriptors did not show significant differences (*p* > 0.05), indicating that critical differential VOCs screened by PLS-DA included most aroma-active compounds in the fermented pea milk samples.

The total ROAVs for each aroma descriptor calculated from the critical differential aroma-active VOCs in the fermented pea milk samples by different starter cultures are displayed as a radar plot in Figure 5C. Compared with the unfermented pea milk, fermentation with different starter cultures changed the deductive sensory characteristics considerably. Levels of fruity, green and fatty odors decreased substantially, while the creamy and sweet odors emerged and grew to substantial levels, and the intensity of pungent odor increased markedly from the near zero level. Among the fermented samples, the creamy, sweet and pungent odors were significantly enhanced by starters 061 and 022, whereas the dairy yogurt starters (465, 485, and 900) exhibited comparatively lower enhancing effects (*p* < 0.05). On the other hand, all starters inhibited the levels of green, fatty and fruity odors, with slightly stronger effects for starters 061 and 022 (*p* < 0.05). The decrease in green and fatty attributes together with the increase in creamy and sweet attributes was consistent with the lower levels of aldehyde-type off-odor VOCs and the higher levels of yogurt-related carbonyl compounds. The stronger creamy, sweet and pungent odors of the 061- and 022-fermented samples were mainly attributed to higher levels of 2,3-butanedione and acetoin, which are formed from α-acetolactate (produced from pyruvate by α-acetolactate synthase) via non-enzymatic oxidative and α-acetolactate decarboxylase-catalyzed decarboxylation, respectively, suggesting more active pyruvate metabolism in these plant-based starters [29]. In addition, the decreases in green, fatty and fruity odors were mainly attributed to the reduction of off-odor aldehydes, such as hexanal and (E)-2-hexenal, to the corresponding alcohols by aldo/keto reductases and alcohol dehydrogenases [22]. Similar changes in volatile profiles after LAB fermentation have been reported in pea milk and soymilk [42,43,44].

As shown in Figure 5D, the deductive sensory characteristics of pea milks fermented by five different starter cultures could be grouped into three clusters. Based on the overall sensory attribute profiles of these clusters, pea milks fermented with plant-based yogurt starter cultures (061 and 022) were assigned to grade 1, those with starters 465 and 900 to grade 2, whereas those with starter 485 were assigned to grade 3. Yogurt samples within the same cluster showed similar sensory characteristics, whereas marked differences in sensory quality were found between clusters, indicating that the deductive sensory characteristics of pea-based yogurt were significantly regulated by different starter cultures.

#### 3.3.2. Supplementation of Carbohydrates

**Figure 6:**
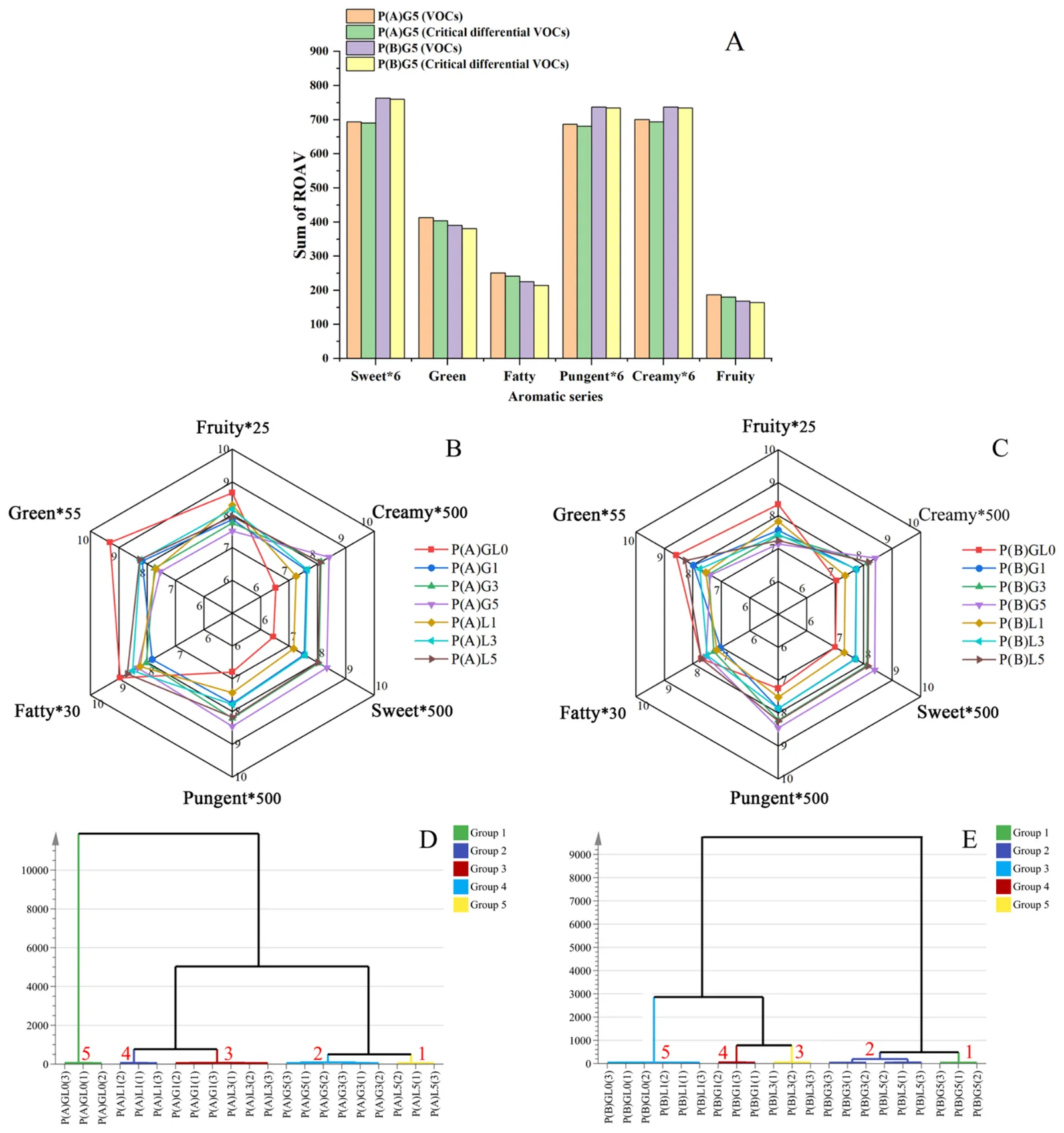
Deductive sensory characteristics of fermented pea milk samples regulated by the addition of different carbohydrates. (**A**) Bar chart of the summed ROAVs of each sensory attribute in P(A)G5 and P(B)G5; the sums were calculated from all VOCs with ROAV > 1 and from the critical differential aroma-active VOCs. (**B**,**C**) Radar plots of the summed ROAVs of the sensory attributes in samples fermented with starters 465 (**B**) and 061 (**C**); the sums were calculated from the critical differential aroma-active VOCs. (**D**,**E**) HCA dendrograms based on the summed ROAVs of the sensory attributes in samples fermented with starters 465 (**D**) and 061 (**E**).

Figure 6A illustrates an example of the comparison between total ROAVs calculated by summing up all VOCs exhibiting ROAV > 1 and by summing up the critical differential aroma-active VOCs in the fermented pea milk samples with 5% added glucose. The two sums did not show significant differences (*p* > 0.05), indicating that the critical differential aroma-active VOCs accounted for most of the aroma-active compounds in fermented pea milk samples.

As illustrated in Figure 6B,C, the deductive sensory characteristics were substantially affected by types and levels of carbohydrates. For the 465-fermented pea milks, the addition of carbohydrates consistently enhanced the desirable creamy odor and reduced the undesirable green odor compared with the unsupplemented sample (*p* < 0.05). More specifically, the creamy odor was remarkably enhanced by 3% and 5% glucose addition, but the least enhancement was observed with 1% lactose addition (*p* < 0.05). With the addition of carbohydrates, the fatty odor was generally weakened, and the lowest fatty intensity was observed when 1% and 3% glucose were added. The enhancing effects on pungent and sweet odors and the inhibitory effect on fruity odor increased with the level of added glucose or lactose. It was found that the green odor was markedly weakened by 3% and 5% glucose and moderately weakened by 5% lactose (*p* < 0.05). The effect of carbohydrate addition on the deductive sensory characteristics of 061-fermented pea milks was similar to that of 465-fermented pea milks, except that the inhibitory effect on fatty odor was smaller. As illustrated in Figure 6D,E, HCA results revealed that the sensory characteristics of fermented pea milks with added carbohydrates could be divided into five groups based on carbohydrate level and type. However, there were differences in the number and types of samples in each group. Based on the overall sensory attribute profiles of the HCA clusters, the starter 061-fermented pea milk samples were classified as follows: 5% glucose (grade 1), 3% glucose and 5% lactose (grade 2), 3% lactose (grade 3), 1% glucose (grade 4), and 1% lactose and zero carbohydrates added (grade 5). These results indicated that the deductive sensory characteristics were substantially affected by the addition of different carbohydrates. These carbohydrate-induced changes were mainly attributed to the increase in 2,3-butanedione and acetoin and the decrease in off-odor aldehydes such as hexanal, suggesting that the added glucose or lactose promoted pyruvate metabolism and the oxidoreductase-mediated biotransformation of these aldehydes [22,29]. Semi-quantification with a single internal standard may bias the ROAVs, and thus the summed ROAVs of the aroma attributes, owing to differences in response factors among VOCs; however, as the VOCs summed for each attribute were largely the same in all samples, its effect on comparisons between samples may be limited. In addition, the odor thresholds used were determined in water rather than in a pea protein matrix, in which protein–VOC interactions may affect the release of VOCs. In future studies, absolute quantification of the critical differential VOCs with authentic standards and determination of their odor thresholds in pea-based matrices would help characterize the deductive sensory characteristics more accurately.

## 4. Conclusions

Starter selection and carbohydrate addition had marked effects on the VOC composition and aroma. Unsupervised overall assessment showed that the volatile components of fermented pea milk samples generally clustered according to the starters used and the levels of carbohydrates added. PLS-DA identified 26 critical differential VOCs in the starter-selection experiment and 27 and 26 in the carbohydrate supplementation experiments with starters 465 and 061, respectively. The up-regulated critical differential VOCs corresponded to the fermentation-generated and biotransformation-generated volatiles, whereas the down-regulated ones were mainly off-odor volatiles in pea milk. To assess the effects of starter selection and carbohydrate addition on the sensory characteristics of fermented pea milks, the ROAVs for each aroma descriptor corresponding to the critical differential aroma-active VOCs were summed up. Because the attribute intensities were calculated from instrumental measurements rather than rated by panelists, this approach revealed the VOCs responsible for the changes in aroma attributes and reduced the influence of subjective factors such as individual perception and experience. The creamy, sweet and pungent odors were markedly enhanced by starters used for plant-based yogurts, whereas dairy-based starters exhibited comparatively lower enhancing effects. On the other hand, all starters inhibited the levels of green, fatty, and fruity odors, with slightly stronger effects for the plant-based yogurt starters. It was found that the effect of glucose or lactose addition on the enhancement of creamy odor and the inhibition of green odor generally depended on the addition levels. HCA showed that deductive sensory characteristics and VOC profiles exhibited generally similar clustering patterns.

## Figures and Tables

**Table 1:** Commercial starter cultures used for pea milk fermentation.

Starter Culture	Application	Declared Microbial Composition
465	Dairy yogurt	*Streptococcus thermophilus*; *Lactobacillus delbrueckii* subsp. *bulgaricus*
485	Dairy yogurt	*Streptococcus thermophilus*; *Lactobacillus delbrueckii* subsp. *bulgaricus*
900	Dairy yogurt	*Streptococcus thermophilus*; *Lactobacillus delbrueckii* subsp. *bulgaricus*
061	Plant-based	*Streptococcus thermophilus*; *Lactobacillus delbrueckii* subsp. *bulgaricus*; *Lacticaseibacillus paracasei*; *Lactobacillus acidophilus* NCFM^®^; *Bifidobacterium animalis* subsp. *lactis* HN019™
022	Plant-based	*Streptococcus thermophilus*; *Lactobacillus delbrueckii* subsp. *bulgaricus*; *Lactiplantibacillus plantarum*; *Lactobacillus acidophilus* NCFM^®^; *Bifidobacterium animalis* subsp. *lactis* HN019™

Note: Specific strain ratios were not disclosed by the manufacturer.

## Data Availability

The data presented in this study are available from the corresponding author upon reasonable request.

## Abbreviations

The following abbreviations are used in this manuscript:

Abbreviation	Full Name
LAB	Lactic acid bacteria
VOCs	Volatile organic compounds
DVS	Direct-vat-set
GC×GC-TOFMS	Comprehensive two-dimensional gas chromatography coupled with time-of-flight mass spectrometry
HPLC	High-performance liquid chromatography
DVB/CAR/PDMS	Divinylbenzene/carboxen/polydimethylsiloxane
RI	Retention index
OAV	Odor activity value
ROAV	Relative odor activity value
PCA	Principal component analysis
PC1, PC2	First and second principal components
HCA	Hierarchical cluster analysis
PLS-DA	Partial least squares discriminant analysis
VIP	Variable importance in projection
ANOVA	Analysis of variance
